# Upgrade Recycling of Cast Iron Scrap Chips towards β-FeSi_2_ Thermoelectric Materials

**DOI:** 10.3390/ma7096304

**Published:** 2014-09-04

**Authors:** Assayidatul Laila, Makoto Nanko, Masatoshi Takeda

**Affiliations:** 1 Graduate School of Engineering, Nagaoka University of Technology, Kamitomioka, Nagaoka, Niigata 940-2188, Japan; E-Mail: laila@stn.nagaokaut.ac.jp; 2 Department of Manufacturing and Materials Engineering, Kuliyyah of Engineering, International Islamic University, Kuala Lumpur 50728, Malaysia; 3 Department of Mechanical Engineering, Nagaoka University of Technology, Kamitomioka, Nagaoka, Niigata 940-2188 Japan; E-Mail: takeda@mech.nagaokaut.ac.jp

**Keywords:** materials recycling, cast iron scrap chips, thermoelectric performance, β-FeSi_2_

## Abstract

The upgrade recycling of cast-iron scrap chips towards β-FeSi_2_ thermoelectric materials is proposed as an eco-friendly and cost-effective production process. By using scrap waste from the machining process of cast-iron components, the material cost to fabricate β-FeSi_2_ is reduced and the industrial waste is recycled. In this study, β-FeSi_2_ specimens obtained from cast iron scrap chips were prepared both in the undoped form and doped with Al and Co elements. The maximum figure of merit (*ZT*) indicated a thermoelectric performance of approximately 70% in p-type samples and nearly 90% in n-type samples compared to β-FeSi_2_ prepared from pure Fe and other published studies. The use of cast iron scrap chips to produce β-FeSi_2_ shows promise as an eco-friendly and cost-effective production process for thermoelectric materials.

## 1. Introduction

Cast-iron is one of the most popular metallic materials used for mechanical components in pipes, machines and automotive parts, such as cylinder heads (declining usage) and block gearbox cases (declining usage). It has become an engineering material with a wide range of applications. Scrap chips of cast-iron were produced from the cutting and milling processes of cast-iron products, which consist primarily of iron with 2.1–4 mass% of carbon and 1–3 mass% of silicon. Thus, cast-iron scrap chips may be suitable starting materials for preparing iron-based materials.

In iron-based materials, β-FeSi_2_ is one of the useful thermoelectric materials at high temperatures. Thermoelectric materials convert temperature differences into electricity and *vice versa*. In the Seebeck effect, a temperature gradient across a material causes the diffusion of charged carriers across the gradient, thus creating a voltage difference between the hot and cold ends of the materials. Semiconducting β-FeSi_2_ has been considered as one of the promising thermoelectric materials, because of its high Seebeck coefficient, low cost, low toxicity and excellent oxidation resistance up to 800 °C [1,2,3]. One great advantage is the fact that both the p-type and n-type of β phase can be instantaneously obtained by doping with Mn or Al [4,5,6] and Co [7,8], respectively. Therefore, both thermocouples can be prepared from the same basic materials, thus eliminating problems of different thermal expansion. In previous studies, the starting materials used to fabricate β-FeSi_2_ were typically pure Fe with 99.99% purity and Si with 99.9% purity [9]. The process of recycling cast-iron scrap chips for β-FeSi_2_, which is an eco-friendly and cost-effective production process, is known as “upgrade recycling”. This is the process of producing high value materials from scraps of low value materials [10]. The material cost is reduced when β-FeSi_2_ is fabricated from the waste of the manufacturing process of cast-iron components. The efficiency of the thermoelectric materials is determined by a dimensionless figure of merit as follows:

*ZT* = *α^2^ σ/k (T)* (1)

where *α* is the Seebeck coefficient; *σ* is the electrical conductivity; and *k* is the thermal conductivity [11]. It is evident from this equation that a large Seebeck coefficient, *α*, a high electrical conductivity, *σ*, and a low thermal conductivity, *k*, are required to obtain a high dimensionless thermoelectric figure of merit, *ZT*.

In this study, the thermoelectric performance of undoped, p-type and n-type β-FeSi_2_ constructed from cast-iron (C.I.) scrap chips is evaluated. Furthermore, the production process of undoped, p-type and n-type β-FeSi_2_ prepared from scrap-chips of C.I. is discussed, *i.e.*, the cleaning process of the cast iron scrap chips, heat treatment, sintering and annealing.

## 2. Experimental Procedure

The cast iron scrap chips were cleaned using ethanol in an ultrasonic bath over 4 cycles of 20 min and then dried in a fume chamber (Yamato Scientific Co. Ltd, Tokyo, Japan). The specimens were then characterized using X-ray fluorescence (XRF) (Rigaku, Tokyo, Japan) and energy dispersion X-ray spectroscopy (EDXS) (EDAX Inc., Mahwah, NJ, USA) for elemental and chemical analyses. The starting materials for undoped, p-type and n-type β-FeSi_2_ were prepared using a solid state reaction of iron grains (99.9% in purity), cast iron scrap chips, silicon grains (99.9% in purity) and powders of the dopant elements: Al (99.9%) for p-type and Co (99.9%) for n-type. The numerical chemical compositions for β-FeSi_2_ prepared from scrap-chips were C.I.:Si = 1:2 and 1:1.86 for undoped samples, C.I.:Co:Si = 0.98:0.02:1.86 for n-type, and C.I.:Si:Al = 1:1.77:0.09 for p-type. For β-FeSi_2_ prepared from pure Fe, the chemical compositions were Fe:Si = 1:2 for undoped samples, Fe:Co:Si = 0.98:0.02:2 for n-type and Fe:Si:Al = 1:1.91:0.09 for p-type. The significance of 1.86 in Si is the chemical composition that needs to be optimized for lesser free silicon after sintering. The powder mixture was prepared using a mortar and planetary ball milling (Asahi Rika Seisakujo, Chiba, Japan) for 1 day. Then, the powder mixture underwent a solid-state reaction at 1100 °C for 3 days in a vacuumed furnace. The reacted powder was then consolidated using a pulsed electric current sintering technique (PECS) (Sinter Land, Niigata, Japan) at 950–1000 °C for 10 min under vacuum conditions and a uni-axial applied pressure of 80 MPa. The sintered samples were finally annealed at 900 °C for 5 days in a vacuumed furnace to obtain the β-FeSi_2_ phase. The sintered samples were characterized using X-ray diffraction (XRD) (Rigaku, Tokyo, Japan) and scanning electron microscopy (SEM) (Keyence, Tokyo, Japan) with EDXS and electron probe micro-analyzer (EPMA) (Shimadzu, Tokyo, Japan). The sintered samples were ground using #600 to #2000 abrasive papers and cut in dimensions of 5 mm × 2 mm × 10 mm for the Seebeck coefficient and electrical conductivity measurement and 12.7 mm in diameter for the electrical property measurements. The electrical conductivity and the Seebeck coefficient for the sintered β-FeSi_2_ were measured using a standard four-probe method and the steady-state temperature gradient along the length of the sample (10 mm) with a commercial apparatus ZEM-2 (Ulvac Co., Tokyo, Japan) at temperatures ranging from room temperature to 800 °C in a stream of He gas. The thermal conductivity was measured from room temperature to 800 °C using a laser flash technique LFA 457 Micro Flash (NETZSCH, Selb, Germany).

**Figure 1:**
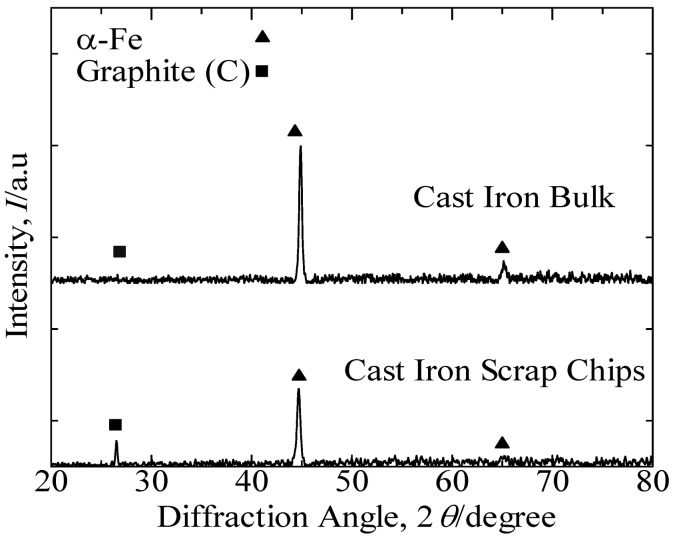
XRD patterns of cast iron scrap chips and cast iron bulk.

**Figure 2:**
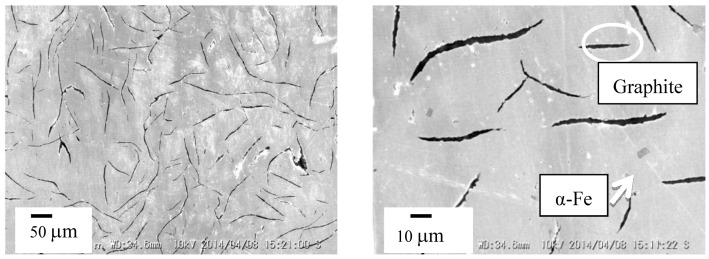
Cross-sectional views of cast iron bulk.

Figure 1 presents the XRD patterns of the cast iron scrap chips and cast iron bulk. These results identified the predominant peak of α-Fe and indicated the graphite peak in both samples. Figure 2 presents the SEM images that reveal the lamellar graphite structure distributed over the α-Fe structure of the cast iron bulk sample. The XRF analysis of the cast iron bulk in Table 1 indicated that the sample consisted primarily of iron with 6.0 mass% of carbon, 2.1 mass% of silicon and minor elements, such as magnesium, manganese, phosphorus and sulfur.

**Table 1:** XRF composition of elements in cast iron bulk.

Element	Mass%
Carbon (C)	6.0
Silicon (Si)	2.1
Minor elements: Mg, Mn, P, S	<0.1
Iron (Fe)	Balance

## 3. Results and Discussion

**Figure 3:**
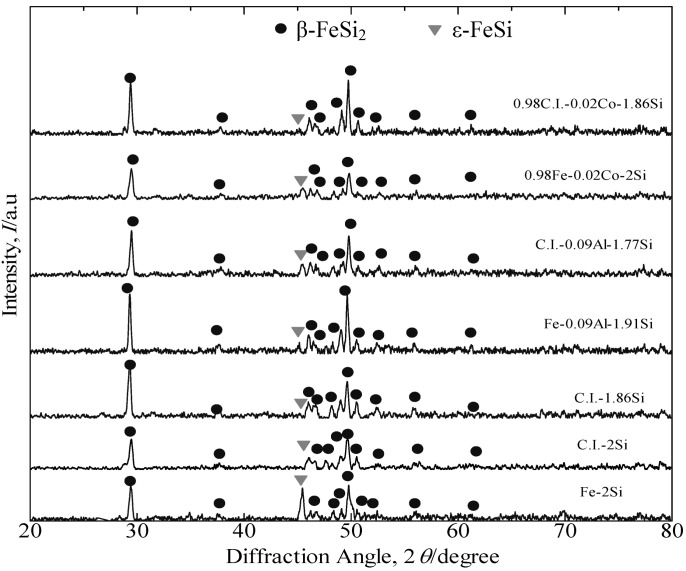
XRD patterns of the annealed β-FeSi_2_ samples at 900 °C for 5 days. C.I., cast-iron; P, pure Fe.

Figure 3 presents the XRD patterns of the annealed β-FeSi_2_ undoped, Co-doped and Al-doped samples prepared from pure Fe and cast iron scrap chips. The prefix “C” presumably stands for “cast iron scrap chips”, which denotes those alloys formed from cast iron scrap chips. Furthermore, the prefix “P” presumably stands for “pure Fe”, which denotes those alloys formed from pure Fe. The dominant peak in all of these samples was β-FeSi_2_. The samples consisted of small amounts of the ε-FeSi phase. The XRD patterns proved that after annealing at 900 °C for 5 days, the simple transition of the peritectoid reaction, α + ε → β, for all samples has been nearly completely transformed to the β phase.

**Figure 4:**
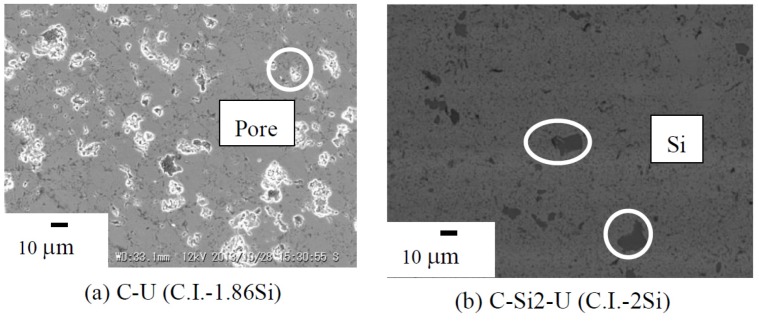
SEM microstructures of the annealed C-U (C.I.-1.86Si) and C-Si2-U (C.I.-2Si) samples at 900 °C for 5 days.

Figure 4 provides the SEM microstructures of the annealed C-U (C.I.-1.86Si) and C-Si2-U (C.I.-2Si) samples at 900 °C for 5 days. By comparing the SEM microstructure of the C-U sample, the excess Si obtained is approximately 20% less compared to that of the C-Si2-U sample. By considering the XRF analysis of the cast iron bulk, the specimen already contained 2.1 mass% of silicon. The C-Si2-U sample consists primarily of β-FeSi_2_ with a slight excess of remaining Si (large black dots). The black dot was determined using electron probe micro-analyzer (EPMA) analysis to be free silicon after sintering. To optimize the numerical chemical composition of Si in the cast iron scrap chips samples, the ratio of powder mixture was reduced from C.I.:Si = 1:2 to C.I.:Si = 1:1.86 for lesser free silicon after sintering. It is necessary to control the composition ratio of C.I.:Si at approximately 1:1.86 to avoid the formation of ε-FeSi in the future. It was reported that the presence of ε-FeSi could arise from a deficiency of Si due to the oxidation during the powder preparation and/or the evaporation during sintering [12]. Additionally, the influence of excess Si in the β-FeSi_2_ specimen prepared from cast iron scrap chips may lead to high thermal conductivity, because the microstructural properties of the materials also have an influence on the materials’ thermoelectric properties.

**Figure 5:**
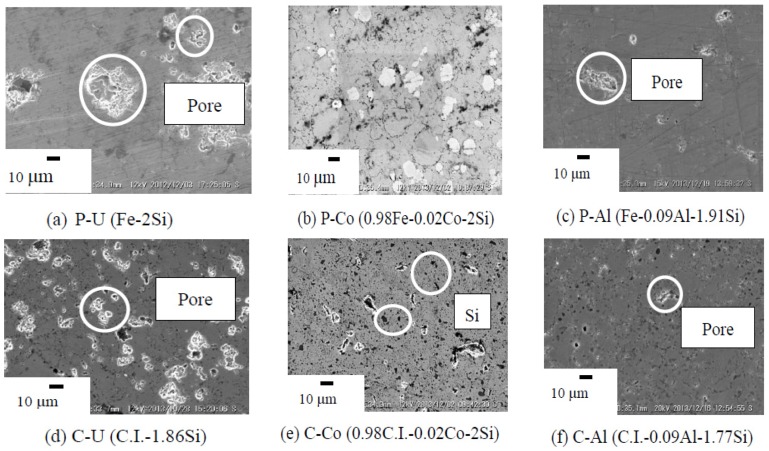
SEM microstructures of the annealed β-FeSi_2_ samples at 900 °C for 5 days.

Figure 5 provides the SEM microstructure for annealed β-FeSi_2_ of undoped, Co-doped and Al-doped β-FeSi_2_ prepared from pure Fe and cast iron scrap chips. As seen in SEM image (e), C-Co were observed in the Si-rich phase [13] (black dot) with small pores (white dot). Several black Si particles detected in the β-phase matrix and pores were scarcely detectable in both the undoped and the Co- and Al-doped samples. The pore size that could be observed from the SEM images was less than approximately 10 µm. Table 2 provides the porosity data of sintered β-FeSi_2_ prepared from pure Fe and cast iron scrap chips. The open porosity observed for all samples was below 1% after sintering, and thus, these were considered as dense samples. For the n-type β-FeSi_2_ prepared from C.I. scrap chips and pure Fe, the Co dopant is definitely substituted for the Fe atom. In the case of the Al-dopant β-FeSi_2_ prepared from C.I. scrap chips and pure Fe, the Al is a p-type dopant and is substituted for the Si atom [14].

**Table 2:** Porosity data for the sintered β-FeSi_2_ samples.

Sample Name (Numerical chemical composition)	Archimedes Measurement
P-U (Fe-2Si)	ρ = 4.7 g/cm^3^, open porosity = 0.3%
C-Si2-U (C.I.-2Si)	ρ = 4.4 g/cm^3^, open porosity = 0.2%
C-U (C.I.-1.86Si)	ρ = 4.7 g/cm^3^, open porosity = 0.4%
P-Co (0.98Fe-0.02Co-2Si)	ρ = 4.7 g/cm^3^, open porosity = 0.2%
C-Co (0.98C.I.-0.02Co-2Si)	ρ = 4.3 g/cm^3^, open porosity = 0.3%
P-Al (Fe-0.09Al-1.91Si)	ρ = 4.4 g/cm^3^, open porosity = 0.5%
C-Al (C.I.-0.09Al-1.77Si)	ρ = 4.5 g/cm^3^, open porosity = 0.3%

**Figure 6:**
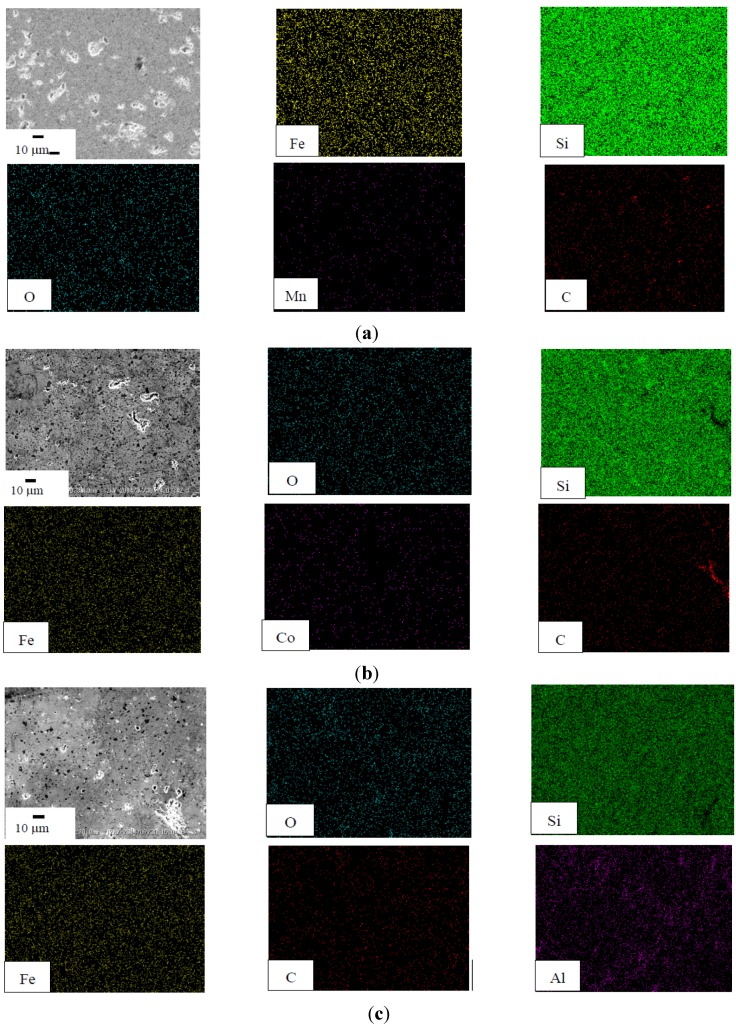
(**a**) SEM image and EDX result of the C-U (C.I.-1.86Si); (**b**) SEM image and EDX result of the C-Co (0.98C.I.-0.02Co-1.86Si); (**c**) SEM image and EDX result of the C-Al (C.I.-0.09Al-1.77Si).

Figure 6a presents the SEM image and the corresponding EDX elemental mapping of the annealed C-U (C.I.-1.86Si) sample. As seen from the mapping images of the EDX, the dominant elements were silicon and iron. By considering the XRD results, this sample was composed of β-FeSi_2_ and had remaining minor elements, such as Mn and C. Figure 6b,c provide the SEM images and the corresponding EDX elemental mapping of the annealed C-Co (0.98C.I.-0.02Co-1.86Si) and C-Al (C.I.-0.09Al-1.77Si) samples, respectively. The distribution of the Co dopant and the Al dopant concentrations in both the n-type and p-type β-FeSi_2_ prepared from C.I. scrap chips was uniformly dispersed in the β-phase.

**Figure 7:**
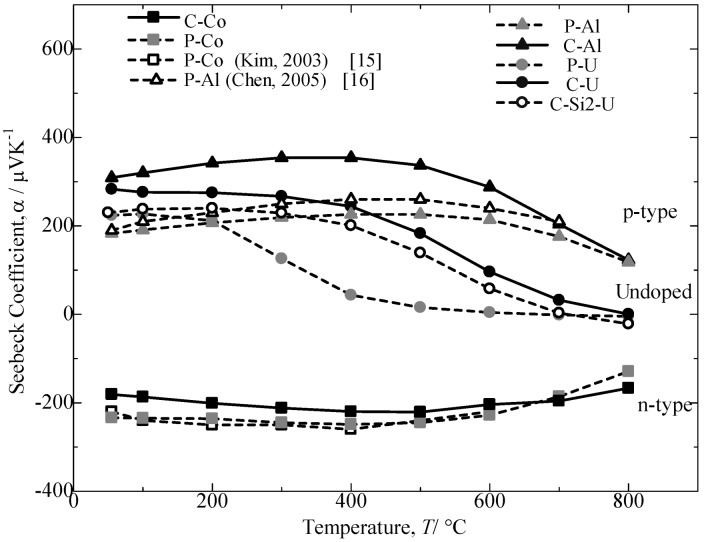
Temperature dependence of Seebeck coefficient, *α*, for the annealed β-FeSi_2_ samples at 900 °C for 5 days.

Figure 7 plots the temperature dependence of the Seebeck coefficient, *α*, for the annealed β-FeSi_2_ samples evaluated from room temperature to 800 °C. In this current study, for the undoped sample, the C-U sample indicates a significantly higher Seebeck coefficient than the C-Si2-U samples. For the doped specimens, it could be observed that different value signs of the Seebeck coefficient are exhibited for the Al-doped and Co-doped β-FeSi_2_. The positive values of the Al-doped samples correspond to the p-type behavior, which indicates that the electrical conductivity is primarily due to holes, while the n-type behavior is attributed to the Co-doped samples from their negative values of the Seebeck coefficient, which indicates electron conduction. The absolute value of the Seebeck coefficient increases with increasing temperature to a maximum and then decreases with a further increase in the temperature. The Seebeck coefficients for the Co-doped and Al-doped β-FeSi_2_ prepared from cast iron scrap chips were obtained at nearly 90% to 100% performance compared to the samples prepared from pure Fe and other reported studies [15,16]. Furthermore, the temperature trend transition and the maximal absolute values for the C-Al and C-Co samples are 400 °C and 354 µVK^−1^ and 500 °C and −221 µVK^−1^, respectively. The preceding increase in *α* could be attributed to a more acute scattering of carriers with an increase in temperature, and the subsequent decrease in the Seebeck coefficient could be due to a rapid increase in the carrier concentration with an increase in temperature [16]. The Seebeck coefficient value for the Al-doping β-FeSi_2_ prepared from cast iron scrap chips was significantly larger compared to that for the Al-doping β-FeSi_2_ prepared from pure Fe. In the Co-doping samples, the difference in values was much smaller.

**Figure 8:**
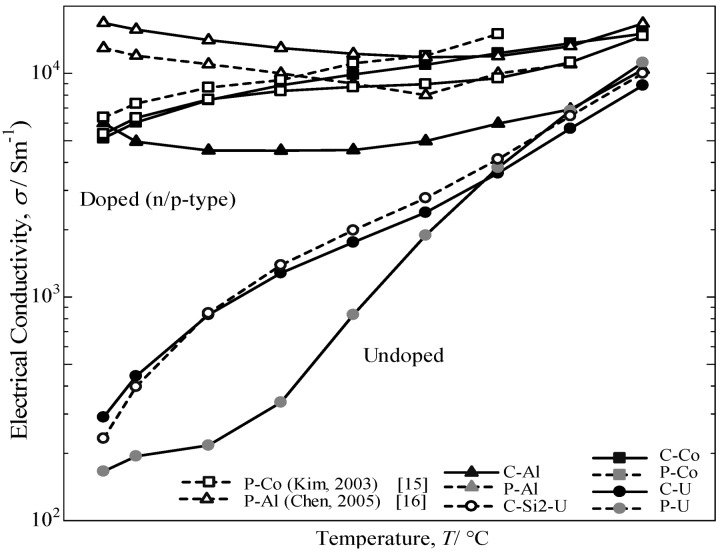
Temperature dependence of electrical conductivity, *σ*, for the annealed β-FeSi_2_ samples at 900 °C for 5 days.

The temperature dependence of the electrical conductivity for the annealed β-FeSi_2_ samples measured from room temperature to 800 °C is provided in Figure 8. The electrical conductivity for the doped samples decreases with increasing temperature up to 500 °C and then increases with a further increase in temperature. Additionally, a sharper increase is noted for the undoped specimen above room temperature. The electrical conductivity of the C-U sample was comparatively the same as the C-Si2-U specimen for undoped samples, but significantly higher than the P-U sample. It was determined that the excess Si in the cast iron scrap chip samples may lead to an increase in the carrier scattering effect of the free Si phase, which is much stronger at lower temperatures than at higher temperatures [2]. Consequently, it is considered that the impurity conductive region for Co-doped and Al-doped β-FeSi_2_ prepared from cast iron scrap chips corresponds to 50–800 °C, and the intrinsic conductive region for undoped β-FeSi_2_ corresponds to 500–800 °C. 

**Figure 9:**
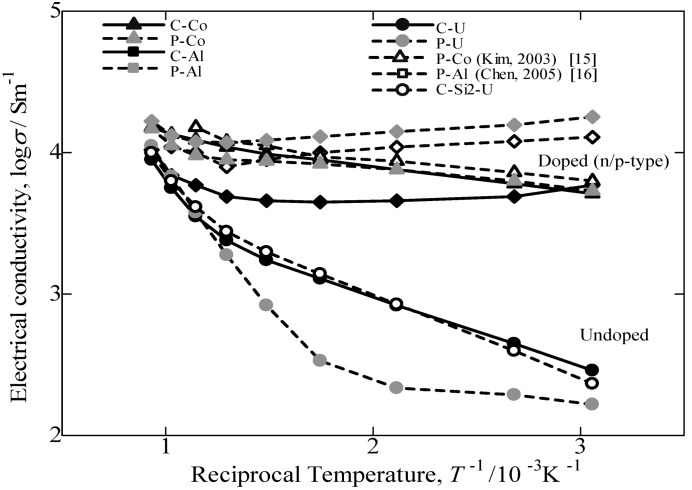
Temperature dependence of electrical conductivity plotted as log *σ vs. T*^−1^/10^−3^K^−1^for the annealed β-FeSi_2_ samples at 900 °C for 5 days.

Figure 9 provides the variation in the logarithm of the electrical conductivity against the reciprocal of the measuring temperature. In this study, compared to the electrical conductivity of others reported in the literature [15,16] and samples prepared from pure Fe, the values of β-FeSi_2_ prepared from cast iron scrap chips are 70% in p-type performance and comparatively the same in n-type performance. Additionally, by comparing the undoped sample prepared from cast iron scrap chips, the obtained electrical conductivity was significantly higher compared to that of pure Fe. The electrical conductivity of the doped samples from both the C.I. scrap chips and pure Fe is nearly constant in the temperature range of RT~800 °C. This behavior is typical for the extrinsic conductive range. Conversely, the value of σ for the undoped samples increases with increasing temperature. This behavior is regarded as intrinsic behavior. Thus, it is confirmed that undoped and Co-doping β-FeSi_2_ prepared from cast iron scrap chips had a positive impact on the electrical conductivity performance compared with others reported in the literature [17,18] and samples prepared from pure Fe. However, in the case of Al-doped β-FeSi_2_ prepared from cast iron scraps, the value of electrical conductivity obtained was lowercompared to that from pure Fe. This finding indicated that the Al doping in the C.I. sample is more sensitive and causes a low carrier concentration effect on the synthesis and the thermoelectric performance. By considering the Seebeck coefficient result, the Al-doping in the C.I. scrap chips also obtained a large difference in value compared to the Al-doping from pure Fe. The reduction in σ is considered to be caused by the ability of the Al-doped in C.I. scrap chips sample to be easily oxidized during sintering, because the C.I. contains numerous impurities. This result is most likely oxidation of the Al-doping by impure oxygen in the cast iron scrap. Thus, the Al-doped β-FeSi_2_ prepared from cast iron scrap obtained a lower performance compared to that of Al-doped β-FeSi_2_ made from pure Fe.

**Figure 10:**
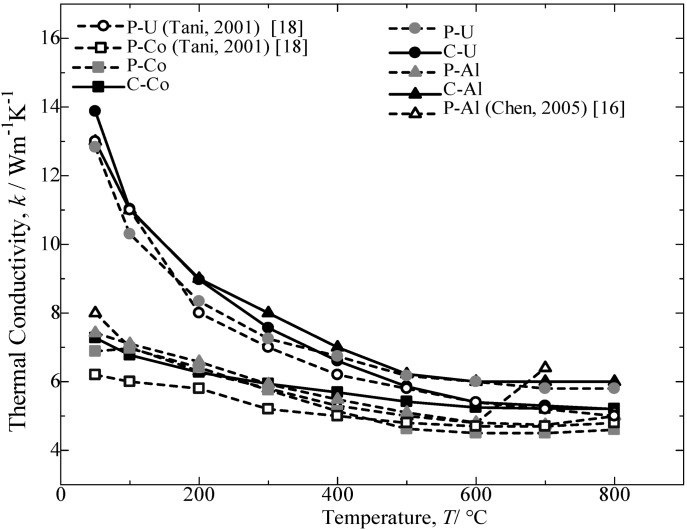
Temperature dependence of thermal conductivity, *k*, for the annealed β-FeSi_2_ samples at 900 °C for 5 days.

Figure 10 provides the thermal conductivity as a function of temperature for the annealed β-FeSi_2_ samples. For all samples, the thermal conductivity decreases with an increase in the temperature. The preceding decrease in thermal conductivity could be attributed to the enhancement of phonon scattering with an increase in temperature [14,19]. The comparison of the thermal conductivity achieved by Al-doped and Co-doped β-FeSi_2_ prepared from C.I. scrap chips with those reported in the literature [16,17] and pure Fe was a 70% (p-type) to nearly a 90% (n-type) performance. Furthermore, the Si-rich in the n-type Co dopant β-FeSi_2_ is reported to improve the transport properties of the materials [18]. A second phase dispersion in the β phase matrix is expected to increase the scattering factors in carriers and phonons, which leads to a higher Seebeck coefficient and a lower thermal conductivity.

**Figure 11:**
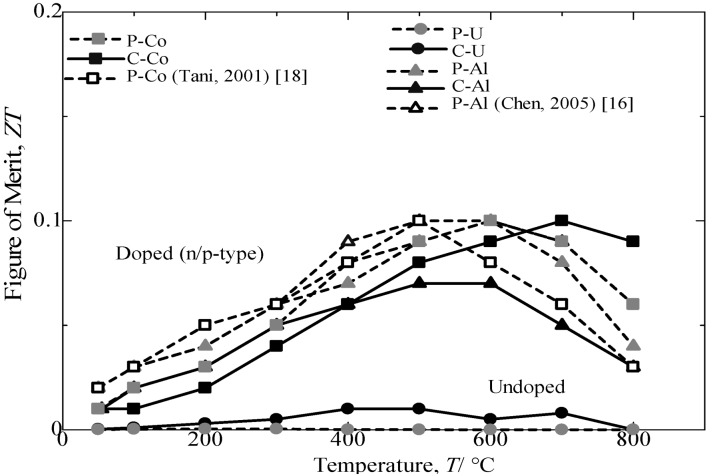
Variation in dimensional figure of merit, *ZT*, with the measuring temperature of the annealed β-FeSi_2_ samples at 900 °C for 5 days.

Figure 11 provides the variations in the dimensionless figure of merit with the measuring temperature of annealed β-FeSi_2_ samples. For all of samples, the *ZT* first increases with an increasing temperature until it reaches the maximum and then decreases with a further increase in temperature. The temperatures for obtaining the maximum *ZT* are 600 and 700 °C for Al-doped and Co-doped β-FeSi_2_, respectively. The variation in *ZT* with measuring temperature resulted from a combination of effects, *i.e.*, changes in the Seebeck coefficient, electrical conductivity and thermal conductivity with measuring temperature [16]. The comparison of the *ZT* achieved from Co-doped and Al-doped β-FeSi_2_ prepared from cast iron scarp chips with those reported in literature [16,17] and pure Fe indicated a good performance in thermoelectric properties at approximately 70% (p-type) and nearly 90% (n-type). The Si-rich in the n-type Co dopant β-FeSi_2_ prepared from cast iron scrap chips was expected to contribute to a higher electrical conductivity and a lower thermal conductivity and, thus, increase the *ZT* [20]. Based on the comparison and discussion, the undoped and the Co-doped β-FeSi_2_ prepared from cast iron scarp chips achieved in the current study are believed to be reasonable and result in comparatively the same performance for β-FeSi_2_ made from pure Fe. However, the Al-doping β-FeSi_2_ prepared from cast iron scarp chips exhibited a low performance in the Seebeck coefficient and electrical conductivity compared with Al-doping made from pure Fe. This effect is due to the high sensitivity to impurity of the Al-doping from C.I. scrap compared with the Co-doping. 

## 4. Conclusions

In the current study, undoped, Co-doped and Al-doped β-FeSi_2_ samples have been successfully synthesized from cast iron scrap chips. By reducing the numerical chemical composition of excess Si in the powder mixture of the C-U sample made from cast iron scrap chips, the thermoelectric performance was significantly enhanced for the C-Si2-U sample compared to that of the sample prepared from pure Fe. The dimensionless figure of merit for the n-type Co dopant and the p-type Al dopant β-FeSi_2_ achieved a 90% and 70% performance, respectively, compared to that of β-FeSi_2_ synthesized from pure Fe and other results published previously. Compared to the Al-doped sample prepared from cast iron scrap chips, the Co-doped sample possesses high electrical conductivity, low thermal conductivity and a high dimensionless figure of merit, *ZT* = 0.1, at a measuring temperature of 700 °C, due to the high carrier concentration effect. The achieved *ZT* is assumed to be comparatively the same for β-FeSi_2_ thermoelectric materials made from pure Fe.
